# Comparing In-Person Only, Telemedicine Only, and Hybrid Health Care Visits Among Older Adults in Safety-Net Clinics

**DOI:** 10.1089/tmr.2023.0003

**Published:** 2023-05-17

**Authors:** Omolola E. Adepoju, Patrick Dang, Melissa R. Valdez

**Affiliations:** ^1^ Department of Health Systems and Population Health Sciences, College of Medicine, University of Houston, Houston, Texas, USA.; ^2^ Humana Integrated Health Systems Sciences Institute, Houston, Texas, USA.; ^3^ Lone Star Circle of Care, Georgetown, Texas, USA.

**Keywords:** telemedicine, older adults, safety-net clinics, access to health care, hybrid appointments

## Abstract

**Introduction:**

Older adults face challenges in seeking health care. This study examined factors associated with in-person only versus telemedicine only versus hybrid health care visits among adults 65+ in safety-net clinics.

**Methods:**

Data were obtained from a large Texas-based Federally Qualified Health Center (FQHC) network. The dataset included 12,279 appointments for 3914 unique older adults between March and November 2020. The outcome of interest was a 3-level indicator of telemedicine visits: in-person visits only, telemedicine visits only, and hybrid (in person + telemedicine) visits during the study period. We used a multinomial logit model adjusting for patient level characteristics to assess the strength of the relationships.

**Results:**

Compared to their white counterparts, black and Hispanic older adults were significantly likely to have telemedicine only visits versus in-person only visits (black RRR: 0.59, 95% confidence interval [CI]: 0.41–0.86; Hispanic RRR: 0.46, 95% CI: 0.36–0.60). However, there were no significant racial and ethnic differences in hybrid utilization (black RRR: 0.91, 95% CI: 0.67–1.23; Hispanic RRR: 0.86, 95% CI: 0.70–1.07).

**Discussion:**

Our findings suggest that hybrid opportunities may bridge racial and ethnic disparities in access to care. Clinics should consider building capacity for both in-person and telemedicine opportunities as complementary strategies.

## Introduction

The COVID-19 pandemic exposed challenges that vulnerable populations seeking health care services, particularly older adults, face. Response to health care access during the pandemic encompassed digital health solutions, such as telemedicine, and its use is projected to increase in the coming years.^1^ Although insurance companies and providers responded rapidly by increasing the scale and scope of coverage of telemedicine services, it is essential that service accessibility remains forefront to ensure universal adoption in vulnerable populations.^2,3^ This is particularly important as telemedicine readiness remains a challenge among older adults citing a lack of technological literacy and auditory and/or visual impairments.^4,5^

Surveys of older adults identified positive reception to telemedicine technologies but suggested that older adults have increased motivation to engage with new technologies when benefits are recognized.^6,7^ This highlights the importance of building on the current telemedicine momentum to promote an equitable future for health care among older adults. Considering that older adults typically spend more on health care services, up to three times their working-age counterparts, they are a highly relevant population for understanding the benefits of telemedicine technologies.^8,9^

Early work suggests that the telemedicine transition for older persons is nuanced, given the unique care needs of older adults. For example, although telemedicine is intended to provide services at the patient's home, nursing and long-term care facilities must also be largely considered.^10–12^ In addition, nine in 10 older adults have one or more chronic diseases, including memory disorders such as Alzheimer's, and dementia.^13,14^ Implementing telemedicine among this patient group will require accommodations and portal optimization.^13^ Evidence suggests that for those in assisted living facilities, telemedicine is effective in augmenting caregiver involvement, optimizing daily rounds between service providers, and reducing the need to travel.^15^

Others have reported significant reductions in emergency department (ED) and in inpatient admits for those living in telemedicine-enhanced acute care facilities.^16^ In primary care, others have suggested that telemedicine may offer significant benefits as it diminishes transportation challenges for persons who require multiple provider visits.^17^

A 2021 survey across the United States reported a steady growth in digital literacy with 96% of those aged 50–64 and 75% of those aged 65+ being internet users, and a corresponding sizable increase from 2010 in use of tablets and other mobile devices.^18^ However, in a study on low-income US older adults receiving care in safety-net clinics, only 53% of participants reported using the internet, while 25% reported having high eHealth literacy.^19^ In central Texas, Choi and Dinitto reported that only 34% of older adults <60 and 17% of those 60 and older self-identified as internet users; and 35% and 16%, respectively, discontinued internet use due to costs and disabilities.^20^ These numbers are even smaller when considering hearing and visual loss, and whether older adults have assistance from digitally literate caregivers, including grandchildren, who can contribute to successful telemedicine consultations.^21^ These highlight the importance of elucidating telemedicine patterns among older adults receiving care in traditionally under-resourced settings, and how these factors could be incorporated in design and user-testing.^22^

Safety net clinics are an important part of the US health care system, providing care to lower socioeconomic status and medically vulnerably populations, including older adults. For many of these clinics, transition to telemedicine during the pandemic not only bridged access to care gaps but also highlighted technological equity challenges for older adults. Understanding factors associated with in-person only, compared to telemedicine only, compared to hybrid health care visits among older adults can elucidate patterns of telemedicine use among populations with greater health care needs such as older adults receiving care in safety net settings. This study examined factors associated with in-person only versus telemedicine only versus hybrid health care visits among older adults receiving care in Texas safety-net clinics.

## Methods

### Data

The primary data source consisted of patient level electronic medical records data obtained from a large safety net clinic network in Texas. Clinic locations span central and south Texas, to include Austin, Houston, Bastrop, Cedar Park, Georgetown, Round Rock, Harker Heights, Hutto, Jonestown, Killeen, Marble Falls, Taylor, and Temple. Data encompassing March–November 2020 patient visits were pulled on all patients 65 or older as of March 2020.

### Measures

The outcome of interest was a 3-level indicator of telemedicine visits: in-person visits only, telemedicine visits only, and hybrid (in-person + telemedicine) during the study period. For the purpose of this study, “in-person only” visits represent individuals who only had in-person visit(s) at one or more Federally Qualified Health Center (FQHC) locations during the study period. Older adults who only had telemedicine visit(s) at one or more FQHC locations during the study period were flagged as having “telemedicine only” visits. Those who had both in-person and telemedicine visits during the study period were flagged as “hybrid.” Independent variables included patient characteristics such as race and ethnicity, age as of March 2020, biological sex, insurance type (private, Medicare, Medicaid, uninsured), and geographic variables: geographic residence in a medically underserved area (MUA), residence in a metropolitan area, and travel time to clinic.

All three geographic variables were included based on empirical literature evidence on their importance for virtual visits. Older adults who had health care consults for acute visits during the study period were flagged having an acute visit; and those who had nonacute health care consults were flagged as having nonacute visits. Accordingly, patients can have both acute and nonacute visits during the study period.

### Model development, evaluation, and analysis

Univariate and bivariate analyses were used to report patient characteristics at the individual patient level. A multinomial logit model adjusting for patient level characteristics was used to compare factors associated with in-person only versus telemedicine only versus hybrid health care visits among older adults receiving care in these safety-net clinics. The model produced relative risk ratios (RRR) and 95% confidence intervals. Importantly, the model captures the decision older adults faced during the pandemic when deciding how to receive healthcare consults. All statistical tests were two-sided, and findings were considered statistically significant at *p* < 0.05. All analyses were conducted using Stata 17.0. This study was approved by an independent Review Board in October 2020.

## Results

### Sample characteristics

Table 1 describes the characteristics of older adults included in the study. The dataset included 12,279 appointments for 3914 unique older adults between March and November 2020. Overall, 53% of older adults in the sample had in-person only visits, 14% had telemedicine only visits, and 34% had hybrid visits. The sample consisted of 35% Non-Hispanic white, 34% Hispanic, and 13% Non-Hispanic black older adults. Majority of the sample (53%) were 65–70 years, 23% were 71–75 years old, 13% were 76–80 years old, and 12% were 81 years or older. Sixty-four percent of the sample were female and ∼96% reside in metropolitan areas. Fifty-six percent of older adults included in this study reside in MUAs.

**Table 1.** Descriptive Characteristics of Older Adults by Patient Appointment Type

Characteristic	Total patients (n = 3914)		Patient appointment type					
			In-person (n = 2057)		Telemedicine (n = 540)		Hybrid (n = 1317)	
	*n*	%	*n*	%	*n*	%	*n*	%
Race/Ethnicity								
White	1386	35.41	729	35.44	250	46.3	470	35.69
Hispanic	1324	33.83	699	33.98	125	23.15	437	33.18
Other	504	12.88	287	13.95	69	12.78	166	12.6
Black	372	9.5	196	9.53	51	9.44	131	9.95
Asian	328	8.38	146	7.1	45	8.33	113	8.58
Age								
65–70	2065	52.76	1125	54.69	288	53.33	652	49.51
71–75	910	23.25	489	23.77	111	20.56	310	23.54
76–80	479	12.24	226	10.99	77	14.26	179	13.59
>80	460	11.75	217	10.55	64	11.85	176	13.36
Sex								
Female	2486	63.52	1198	58.24	372	68.89	916	69.55
Male	1428	36.48	859	41.76	168	31.11	401	30.45
Metropolitan area residence								
Metropolitan	3736	95.53	1939	94.36	522	96.67	1275	96.88
Non-Metropolitan	175	4.47	116	5.64	18	3.33	41	3.12
MUA residence								
MUA	2177	55.66	1146	55.77	318	58.89	713	54.18
Non-MUA	1734	44.34	909	44.23	222	41.11	603	45.82
Insurance coverage								
Medicare	2522	64.45	1319	64.15	344	63.7	859	65.22
Uninsured	1004	25.66	508	24.71	127	23.52	369	28.02
Private	311	7.95	184	8.95	52	9.63	75	5.69
Medicaid	76	1.94	45	2.19	17	3.15	14	1.06
Travel time from FQHC Clinic								
0–10 min	1155	29.51	621	30.19	158	29.26	376	28.55
11–20 min	1566	40.01	818	39.77	209	38.7	539	40.93
21–30 min	821	20.98	435	21.15	120	22.22	266	20.2
>30 min	372	9.5	183	8.9	53	9.81	136	10.33
Appointment reason/visit type								
Acute	2518	64.33	1083	52.65	312	57.78	1123	85.27
Others (non-acute visit, screenings/examinations)	1396	35.67	974	47.35	228	42.22	194	14.73

FQHC, Federally Qualified Health Center; MUA, medically underserved area.

Approximately 65% of the sample had Medicare coverage, 8% had private health insurance coverage, 2% had Medicaid as their primary insurance, and 26% were uninsured. Geographically, 96% of the sample resided in a metropolitan area while 56% resided in MUA. For about 70% of the sample, average travel time from patient residence to provider clinic was 20 min or less.

Table 2 reports the multinomial regression model comparing in-person visits only, telemedicine visits only, and hybrid visits. Compared to their non-Hispanic white counterparts, non-Hispanic black and Hispanic older adults were significantly less likely to have telemedicine only visits versus in-person only visits (black RRR: 0.59; 96% confidence interval [CI]: 0.41–0.89; Hispanic RRR: 0.47, 95% CI: 0.36–0.60). While increasing age was not significantly associated with telemedicine visits, males were significantly less likely to have telemedicine only versus in-person only visits (RRR: 0.60, 95% CI: 0.49–0.74).

**Table 2.** Multinomial Logit Regression Model Comparing in-Person Visits Only, Telemedicine Visits Only, and Hybrid (in Person + Telemedicine) Visits

Variables	MV adjusted RRR		
	RRR	95% CI	*p*
In person only	(base outcome)		
Telemedicine only			
Race/Ethnicity			
NH white	Ref		
NH black	0.59	0.41–0.86	0.006
Hispanic	0.47	0.36–0.60	<0.001
Asian	1.21	0.86–1.72	0.278
Other	0.51	0.36–0.73	<0.001
Age			
65–70	Ref		
71–75 years	0.91	0.71–1.18	0.488
76–80 years	1.32	0.97–1.80	0.073
>80 years	1.28	0.93–1.77	0.135
Sex			
Female	Ref		
Male	0.60	0.49–0.74	<0.001
Metropolitan area residence			
Nonmetro	Ref		
Metro	2.14	1.23–3.72	0.007
MUA residence			
Non-MUA	Ref		
MUA	1.01	0.81–1.24	0.957
Insurance type			
Private	Ref		
Medicaid	1.90	0.96–3.74	0.065
Medicare	0.93	0.66–1.32	0.684
Uninsured	0.99	0.67–1.45	0.940
Time			
0–10 min	Ref		
11–20 min	0.93	0.73–1.18	0.550
21–30 min	1.02	0.77–1.36	0.883
>30 min	1.29	0.88–1.88	0.197
Had nonacute visit	1.22	0.91–1.64	0.189
Had acute visit	1.02	0.91–1.50	0.054
Travel time			
March	0.19	0.13–0.27	<0.001
April	1.67	1.32–2.10	<0.001
May	1.31	1.06–1.62	0.013
June	0.93	0.77–1.12	0.431
July	1.20	1.00–1.43	0.051
August	0.75	0.61–0.92	0.006
September	0.68	0.55–0.84	<0.001
October	0.64	0.52–0.79	<0.001
November	0.61	0.49–0.76	<0.001
Hybrid visits			
Race/Ethnicity			
NH white	Ref		
NH black	0.91	0.67–1.23	0.551
Hispanic	0.86	0.70–1.07	0.174
Asian	1.29	0.92–1.79	0.138
Other	0.77	0.58–1.02	0.066
Age			
65–70	Ref		
71–75 years	1.09	0.89–1.34	0.412
76–80 years	1.31	1.00–1.70	0.047
>80 years	1.58	1.21–2.06	0.001
Sex			
Female	Ref		
Male	0.65	0.55–0.78	<0.001
Metropolitan area residence			
Nonmetro	Ref		
Metro	1.82	1.15–2.90	0.011
MUA residence			
Non-MUA	Ref		
MUAs	0.98	0.82–1.17	0.786
Insurance type			
Private	Ref		
Medicaid	0.47	0.21–1.03	0.059
Medicare	1.23	0.88–1.72	0.221
Uninsured	1.15	1.02–2.06	0.079
Travel time			
0–10 min	Ref		
11–20 min	1.28	1.05–1.58	0.017
20–30 min	1.10	0.86–1.40	0.458
>30 min	1.69	1.23–2.32	0.001
Had nonacute visit	2.84	2.29–3.52	<0.001
Had acute visit	1.10	0.91–1.58	0.061
Time			
March	1.39	1.21–1.59	<0.001
April	2.58	2.15–3.08	<0.001
May	2.15	1.83–2.52	<0.001
June	1.32	1.16–1.51	<0.001
July	1.55	1.36–1.76	<0.001
August	1.20	1.05–1.37	0.008
September	1.18	1.03–1.35	0.014
October	1.11	0.98–1.26	0.114
November	1.25	1.09–1.43	0.002

CI, confidence interval; MV, multivariable; NH, non-Hispanic; RRR, relative risk ratios.

Compared to those residing in nonmetropolitan areas, older adults residing in metropolitan areas were significantly more likely to have telemedicine only visits versus in-person only visits (RRR: 2.14, 95% CI: 1.23–3.72). Telemedicine only visits were less likely to occur in the later months of 2020 (August–November) in line with the relaxation of the stay-at-home orders. Insurance type was not associated with telemedicine only versus in-person only visits.

There were no significant racial and ethnic differences in hybrid visits versus in-person only visits (black RRR: 0.91; 96% CI: 0.67–1.23; Hispanic RRR: 0.86 95% CI: 0.70–1.07). Compared to older adults 65–70 years, those 76–80 years (RRR: 1.31, 95% CI: 1.00–1.70) and >80 years (RRR: 1.58, 95% CI: 1.21–2.06) were more likely to have hybrid visits versus in-person only visits. Males were significantly less likely to have hybrid visits versus in-person visits (RRR: 0.65, 95% CI: 0.55–0.78). Older adults with nonacute visits concerns were more likely to have hybrid visits versus in-person visits (RRR: 2.84, 95% CI: 2.29–3.52).

Compared to those residing in nonmetropolitan areas, older adults residing in metropolitan areas were significantly more likely to have hybrid visits versus in-person visits (RRR: 1.82, 95% CI: 1.15–2.90). Travel time from a clinic was associated with an increased likelihood hybrid visits, and patients with travel times >30 min exhibited the greatest likelihood (RRR: 1.69, 95% CI: 1.23–2.32). With the exception of October 2020, hybrid visits were consistently higher throughout the pandemic months (March to November), compared to in-person only.

## Discussion

To the best of our knowledge, this is one of the few studies to examine factors associated with in-person only versus telemedicine only versus hybrid health care visits among older adults receiving care in safety-net clinics. Because safety net clinics serve primary and behavioral health care needs of all individuals regardless of insurance status or ability to pay, this research focuses on a doubly vulnerable and underserved population, typically at greater risks for suboptimal health outcomes. Importantly, our findings highlight older adults' use of in-person and telemedicine visits as complements and that hybrid opportunities might bridge disparities in access to care gaps following no significant racial and ethnic differences in hybrid utilization.

After adjusting for the month a visit occurred, this study observed differences in the use of telemedicine based on phases of the COVID pandemic. Older adults were relatively more likely to have telemedicine only visits between April and July 2020, compared to in-person only visits, and vice-versa between August and November 2020. We posit that this may be attributable to stay at home orders, which were lifted around mid/late summer 2020. Importantly, this finding is consistent with previous reports on the impact of COVID-19 on outpatient visits. For instance, one report found that in April 2020, there was a 58% decrease in-person visits from the baseline, and a corresponding increase of 12.5% for telemedicine.^23^

We also found that hybrid visits were significantly more likely to occur during the entire study (March–November 2020), for both acute and nonacute visits, suggesting the need for clinics to build capacity to provide both more in-person and telemedicine opportunities as complementary strategies to continue patient care. This finding is of significance because 85% of individuals 65 or older have at least one chronic condition, while 60% have at least two.^24^ Many of these chronic conditions affect their mobility, vision, and/or hearing.^25^ These challenges ultimately make it difficult for many older adults to have frequent in-person visits to monitor their chronic conditions.^25^ While telemedicine may reduce the need for frequent in-person visits, the use of telemedicine portals with accessibility customization is an important consideration in this population.^26^

Efforts toward technological equity include improving telemedicine programs to accommodate individuals with age-related sensory and cognitive impairments by actively including them in user-testing, and providing affordable tools and internet services for older adults.^27^

Older adults residing in a metropolitan area were more likely to have telemedicine and hybrid visits during the pandemic. This aligns with findings from a National Health and Aging Trends Study that examined telemedicine unreadiness among older adults, and reported significantly greater readiness among those who resided in metropolitan areas.^4^ Greater travel time to the clinic also contributed to a higher likelihood of hybrid visits, aligning with earlier work that found that the distance from the hospital was a good indicator of whether a patient would be likely to use telemedicine as opposed to going to the hospital for a visit.^28^ This finding uncovers certain challenges because individuals who live in rural areas are more likely to live further away from a clinic but also less likely to have broadband internet access.^29^ From an access to care perspective, this finding has significant implications for nonmetropolitan and/or rural-dwelling older adults.

Compared to non-Hispanic whites, racial and ethnic minority older adults were less likely to have telemedicine visits. This is somewhat in contrast to a 2021 study that examined gender, racial, and ethic disparities in telemedicine and reported lower odds for telemedicine use in Hispanic patients (vs. non-Hispanic patients), and higher odds for telemedicine use among black patients (vs. white patients).^30^ Importantly, there were no significant racial and ethnic differences in hybrid visits suggesting hybrid opportunities might bridge disparities in access to care gaps. Age also strongly determined the use of hybrid visits. Compared to older adults aged 65–70, persons 76–80 and >80 were more likely to be hybrid users, contrasting with previous work on telehealth use among older adults during the pandemic, where increasing age was associated with a lower likelihood of receiving virtual care.^31^

While this study has several strengths, the following limitations should be considered when interpreting these results. Our analyses use data from safety-net clinics, and therefore, these findings may not be representative of other types of clinics across the country. There are also various challenges in widespread telemedicine adoption for safety-net clinics that are not fully addressed in this study, such as broadband internet access, usability, or patient preference.^32^ While approximately half of American older adults own a smartphone, and two-thirds use the internet, accessibility and experience with using newer technology varies according to income and education.^33^ This contributes to difficulties with navigating the telemedicine portals and dealing with in-the-moment connectivity and technological issues.^25^

Some studies suggest that one of the main predictors of whether older adult patients will continue to use telemedicine is their perceived autonomy when using the service, as well as how connected they felt to the providers they were speaking with.^34^ Therefore, developing devices and applications where modifications can be made is key. Finally, this study also occurred during the initial/peak phase of the COVID-19 pandemic that was characterized by significant disruptions to the health care system, and this limits the generalizability of our study findings in a postpandemic period.

In conclusion, recognizing determinants of disparate telemedicine adoption provides an opportunity for targeted interventions to increase telemedicine use among older adults. This promotion of hybrid visits offers immense potential that could close racial and ethnic differences in access to care.

## Abbreviations Used

CI: confidence interval
FQHC: Federally Qualified Health Center
MUA: medically underserved area
MV: multivariable
NH: non-Hispanic
RRR: relative risk ratios
